# Time Perception in Prodromal Alzheimer's Dementia and in Prodromal Dementia With Lewy Bodies

**DOI:** 10.3389/fpsyt.2021.728344

**Published:** 2021-10-07

**Authors:** Ming-Chyi Pai, Chiu-Jun Yang, Sheng-Yu Fan

**Affiliations:** ^1^Institute of Gerontology, College of Medicine, National Cheng Kung University, Tainan, Taiwan; ^2^Division of Behavioral Neurology, Department of Neurology, College of Medicine, National Cheng Kung University Hospital, National Cheng Kung University, Tainan, Taiwan; ^3^Alzheimer's Disease Research Center, National Cheng Kung University Hospital, Tainan, Taiwan

**Keywords:** prodromal Alzheimer's dementia, prodromal dementia with Lewy bodies, time perception, time estimation, accuracy, precision

## Abstract

**Background:** Time perception is a subjective experience or sense of time. Previous studies have shown that Alzheimer's dementia (AD) patients have time perception deficits compared to a cognitively unimpaired control group (CU). There are only a few studies on dementia with Lewy bodies (DLB) patients' time perception in comparison with CU and AD patients. Early intervention and prescription of the right medicine may delay the deterioration of AD and DLB, moreover, knowing how prodromal AD (prAD) and prodromal DLB's (prDLB) time perception differ from each other might be helpful for future understanding of these two dementias. Therefore, the purpose of this study is to explore the difference in time perception performance between prodromal AD and prodromal DLB.

**Methods:** We invited people diagnosed with prAD, prDLB, and CU to participate in this study. Tests of verbal estimation of time and time interval production were used to assess their time perception. We analyzed the average time estimation (ATE), absolute error score (ABS), coefficient of variance (CV), and subjective temporal unit (STU) within the three groups.

**Results:** A total of 40 prAD, 30 prDLB, and 47 CU completed the study. In the verbal estimation test, the CV for the prAD was higher than both prDLB and CU at the 9 s interval, and the CV of prAD was higher than CU at the 27 s interval. In the time interval production test, the subjective time units of prDLB were higher than prAD at the 10 s interval, while those of both prDLB and CU were higher than prAD at the 30 s interval. The percentage of subjects with STU < 1.0 s, indicating overestimation, was higher in prAD than both prDLB and CU.

**Conclusion:** Time perception of prAD patients showed imprecision and overestimation of time, while prDLB tended to underestimate time intervals. No significant difference was found in accuracy among the three groups. It is speculated that the clinical and pathological severity of the two prodromal dementia stages may be different, and some patients have not yet had their time perception affected.

## Introduction

Time perception is an extremely complex and indispensable function for humans involving many cognitive processes. It allows us to carry out various behaviors on different time scales, such as making tea, catching a bus or train, setting flight plans, and so on (1). Researchers studied people with neurological disorders to elucidate the relationship between the performance of time perception and specific brain areas. They have found that the hippocampus, entorhinal cortex, prefrontal lobe cortex, insula, cerebellum, and basal ganglia are related to time perception, though the mechanisms involved remain somewhat unclear (1–11).

Perhaps the most plausible model of timing in the psychological process is the pacemaker-accumulator model (11, 12). In this model, time perception is divided into three stages, namely, clock, memory, and decision and it assumes that time perception originates from the pacemaker, and that the pacemaker produces pulses. The pulses pass through a switch and enter an accumulator. The pulses collected by the accumulator transform into a specific subjective time interval which is stored in short term memory first and subsequently long-term memory. When the next pulse turns into a sense of time, the subjective time interval previously stored in long term memory is retrieved and compared with the time interval in short term memory. The subjects determine the length of the two time intervals, affecting corresponding behaviors (5).

In addition to physical and psychological mechanisms, factors such as attention and emotion may influence time perception as well. Researchers believe that the greater the complexity of the test, the more attention needed to deal with it. They also believe that attention plays a role in controlling the switch in the pacemaker-accumulator. When paying attention, the switch may need to generate many pulses. Another factor is emotion. Positive and negative valence of the people's emotions, as well as their level of arousal, may influence the feeling of time passed. Even though emotion can be a powerful regulator for time perception, the neural mechanism of time distortion caused by emotion is still unclear. In an experiment, when the degree of arousal was high, negative valence resulted in a time interval perception longer than positive valence did, however, when the degree of arousal was low, the opposite result obtained (5, 13).

Alzheimer's dementia (AD) and dementia with Lewy bodies (DLB) are the most two common primary degenerative dementias. Time perception in people living with AD is different from that in people of the same age and same education level (1, 14), although the differences may arise from different tests used. A few studies in this field have been done in DLB and revealed that performance on the rhythm test and verbal estimation (VE) test was inferior to a normal control group of the same age (15). As is known, people with DLB have a clinical symptom of fluctuating cognition, and in the later stages, memory deficit may also occur (16). Since time perception involves attention and memory (5), a study of time perception in DLB is clinically important. Moreover, in recent years, researchers have been interested in the prodromal stage of AD and DLB and have conducted research hoping to develop a reliable method to identify AD and DLB and to conduct early interventions to slow down the deterioration of their clinical symptoms. Since impaired episodic memory and cognitive fluctuation are the hallmarks of clinical manifestation for each type of dementia, it is plausible to suggest an impaired time perception in their prodromal stages. The aim of this study was to investigate the difference of time perception in prodromal AD dementia (prAD) and prodromal dementia with Lewy bodies (prDLB).

## Materials and Methods

### Participants

People with prAD and prDLB were recruited from a special dementia clinic of a national university hospital located in southern Taiwan. We also invited cognitively unimpaired (CU) participants either from the special clinic or patient's spouses. The study was approved by the Institutional Review Board of National Cheng Kung University Hospital and all participants signed informed consent before the experiments.

The inclusion criteria for a diagnosis of prAD were as follows: (1) SPECT showed decreased perfusion in parietal association area, posterior cingulate, precuneus; (2) magnetic resonance imaging (MRI) showed atrophy, especially in the medial temporal lobe and/or posterior cortical regions; (3) objective evidence of impairment in one or more cognitive domains, including memory, attention, language, visuospatial skills, or executive function; (4) independent activities of daily living; (5) other conditions that may cause cognitive impairment must be ruled out (17–19). The inclusion criteria for the diagnosis of prDLB were as follows: (1) more than one core symptoms of DLB, such as fluctuating cognition, visual hallucinations, rapid eye movement sleep behavior disorder (RBD), and more than one symptom of Parkinson's disease; (2) using TRODAT to confirm the reduced uptake of dopamine transporter in striatum; (3) independent activities of daily living; (4) other conditions that may cause cognitive impairment must be ruled out (20, 21).

To eliminate deviation of the results caused by improper use of the tablet computer, people with illiteracy, or a speech or language impairment, were excluded. Similarly, people with a history of other non-AD or non-DLB related neurological disorders (such as epilepsy, stroke, encephalitis, and head trauma) were also excluded.

### Assessments

A battery of neuropsychological tests was administered to all participants before the time perception test started. The Cognitive Ability Screening Instrument (CASI) was used as a tool to determine the general cognitive function of the participants. The Clinical Dementia Rating (CDR) Scale was used as an assessment for daily function. Neuropsychiatric Inventory (NPI) was mainly to assess emotional, behavioral, psychological, and other non-cognitive symptoms.

**Figure 1:**
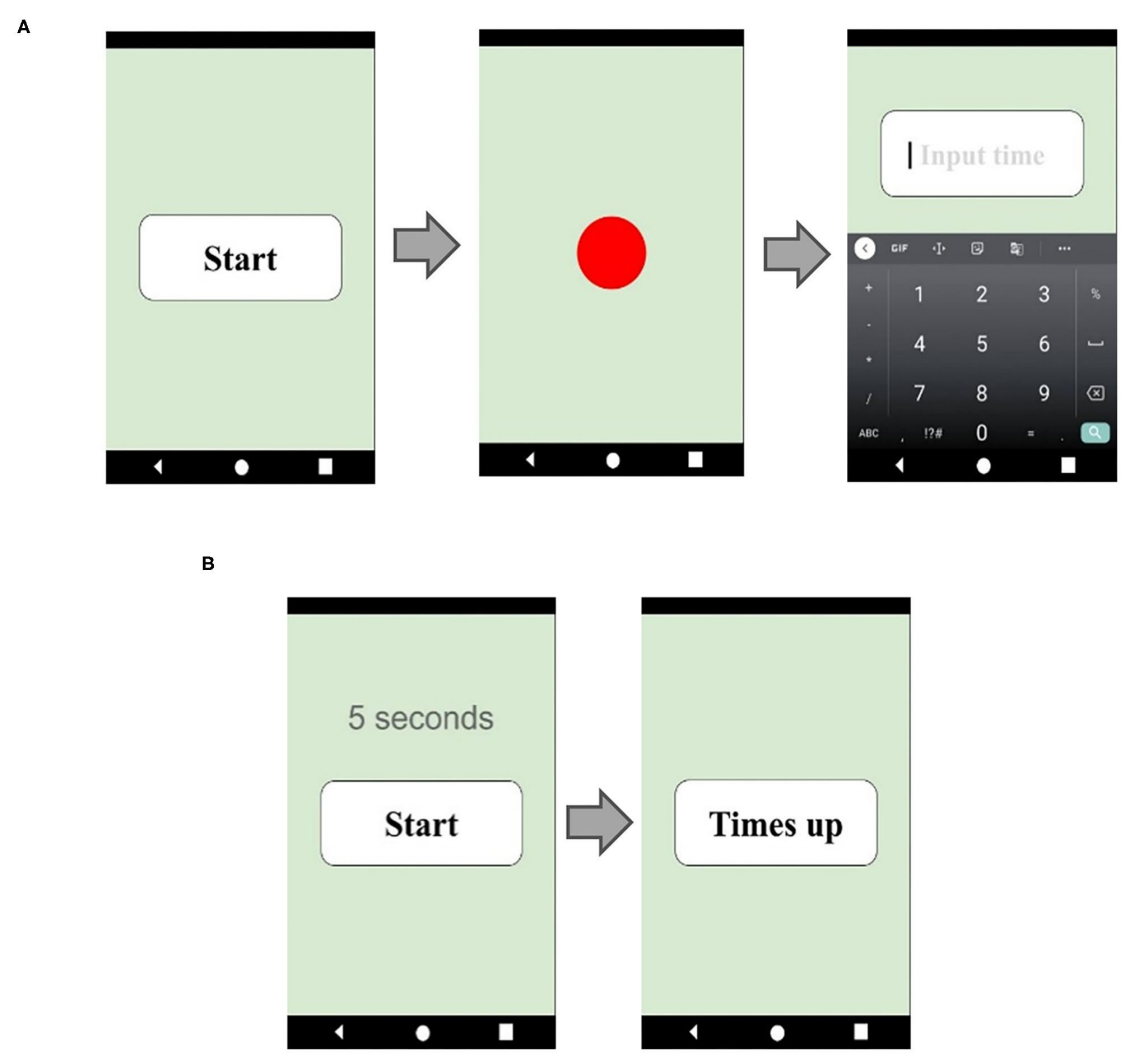
**(A)** Verbal estimation test. **(B)** Time interval production test.

To evaluate time perception, all participants were asked to complete two different prospective time perception tests: a verbal estimation (VE) test and a time interval production (TP) test. Time perception tests use tablet software as the main test tool (Figure 1). The VE test was administered first. We asked the participants to take a deep breath and calm down, and then had them press the start button at will. When the button was pressed, a red circle immediately lit up indicating the start of the target time interval. When the red circle disappeared, indicating the target interval was over, an input panel appeared on the screen, and the participants were asked to enter how long the red circle had been on the screen. The instructions were: “*Please press the button when you are ready, a red circle will appear on the screen immediately. Please estimate how many seconds the red circle was on the screen and enter your estimate*.” A total of 11 trials was performed. At first, two practice trials, with 5 s time interval, were given so that the participants could become familiar with the test, and it acquainted us that the participants had understood the directions sufficiently. Moreover, the practice trials may also have had a calming effect on the participants, making it easier for them to concentrate. The remaining 9 trials with target time intervals 9, 27, and 56 s, three times for each, appeared randomly, and the participants' estimations were recorded on the tablet, see Figure 1A. The reason why we used 9, 27, and 56 s instead of 10, 30, and 60 s was to avoid the possibility that the participants may guess.

The TP test followed the VE test. We asked the participants to produce a target interval. The participants were taught to take a deep breath and calm down, press the start button on the tablet, start counting the seconds silently, and immediately press the “time up” button when the target number of seconds had been counted. The software automatically recorded the results. The instructions were: “*Please press the button when you are ready and press the “time up” button when you think you have reached the target number of seconds*.” Once again, two practice trials were given. The remaining nine trials' target time intervals were 10, 30, and 60 s, each target time interval appeared three times randomly, and the tablet software automatically recorded the elapsed time, see Figure 1B.

### Statistical Analysis

To verify the demographic variables of the three groups, a chi-square test was used to test their gender. One-way ANOVA tests were applied to see the differences in age, years of education, neuropsychological tests, and the NPI.

Average time estimation (ATE), absolute error values (ABS), coefficient of variation (CV), and the subjective temporal unit (STU) were derived from the VE and the TP tests. The ABS were the deviations in seconds a participant made from the target intervals. ATE and ABS are considered the overall accuracy level of the time estimation test. CV is the result of dividing the standard deviation by the average time estimate, which evaluates the precision of the time estimation test. STU is calculated by dividing the target interval by the participants' estimate, indicating over- or underestimation of time interval. For STU, a score greater or <1.0 s indicates an under- or overestimation (22–24). These four variables were examined using one-way ANOVA, and the group was used as an independent variable to see the accuracy, precision, and over-/underestimation in different time intervals. A Chi-square test was performed to analyze the differences in the proportion of people who were either over- or underestimating among the three groups.

Statistical analyses were performed using SPSS for Windows (SPSS 17; SPSS Inc.). *Post-hoc* Scheffe tests were performed to reveal the differences between pairs. An alpha level of 0.05 was set in all tests.

## Results

A total of 146 participants signed an informed consent form. Among them, 25 withdrew later for fear of the SARS-Cov-2 pandemic, two were excluded because of an inability to follow the instructions, and two failed to finish the whole task. One-hundred and seventeen participants completed the study, including 47 CU, 40 prAD, and 30 prDLB. Tables 1, 2 shows the demographics and neuropsychological test results. A difference was detected in gender and age among the three groups. After reran statistical analysis using Pearson correlation with these variables, however, we found a low correlation between age and gender and the outcomes (ATE, ABS, CV, and STU). PrAD had a higher proportion of using cholinesterase inhibitor than prDLB. It might be due to Taiwan National Health Insurance Regulations, i.e., cholinesterase inhibitor medication is approved and reimbursed for prAD rather than prDLB patients, and some prDLB patients would buy it with their pocket money.

**Table 1:** Demographic characterization and CASI.

**Variables**	**Total (%)**	**CU**	**prAD**	**prDLB**	***x*^**2**^/*F***	***p*-value**	***post-hoc***
		**(*****n*** **=** **47)**	**(*****n*** **=** **40)**	**(*****n*** **=** **30)**			
Sex (M/F)^a^	52/65	19/28	12/28	21/9	11.62	0.003^**^	
Age (years)^b^	71.74 (7.29)	69.70 (8.22)	73.43 (5.85)	72.70 (6.91)	3.29	0.041^*^	
Education (years)^b^		12.17 (3.43)	10.75 (3.89)	12.90 (4.34)	2.93	0.058	
Using ChE I^a^		0 (0%)	34 (85%)	13 (43%)	65.12	0.000^***^	prAD > prDLB > CU
CDR (SoB)^b^	1.00 (1.06)	0.14 (0.31)	2.05 (0.90)	0.95 (0.75)	86.80	0.000^***^	prAD > prDLB > CU
**CASI** ^**b**^
Remote memory		10.00 (0.00)	9.75 (0.67)	9.97 (0.18)	4.56	0.012^*^	A
Recent memory		10.78 (1.73)	5.44 (2.69)	10.17 (1.99)	73.17	0.000^***^	B
Attention		7.61 (0.88)	6.85 (1.31)	7.13 (1.14)	5.12	0.007^**^	A
Mental manipulation		9.33 (1.37)	8.33 (1.93)	8.97 (1.65)	3.98	0.021^*^	A
Orientation		17.87 (0.45)	11.63 (3.76)	16.73 (1.93)	76.40	0.000^***^	B
Abstract thinking		10.12 (1.74)	9.05 (1.87)	9.80 (1.81)	3.81	0.025^*^	A
Language		9.74 (0.61)	9.30 (1.04)	9.40 (0.72)	3.44	0.036^*^	A
Drawing		9.91 (0.34)	8.95 (2.11)	9.27 (1.64)	4.55	0.013^*^	A
Animal		8.67 (1.94)	6.08 (2.37)	7.53 (1.83)	16.80	0.000^**^	B
CASI total score		94.17 (5.43)	75.49 (10.82)	89.40 (6.79)	61.10	0.000^***^	CU > prDLB > prAD

*Data are presented as mean (standard deviation) or number (percentage). Post-hoc analysis by Scheffe's test*.

*CU, cognitively unimpaired; prAD, prodromal AD; prDLB, prodromal DLB; ChE I, cholinesterase inhibitors; CDR SoB, Clinical Dementia Rating Scale, Sum of Box; CASI, Cognitive Abilities Screening Instrument*.

*A, CU > prAD; B, CU = prDLB > prAD; a, Using Chi-square; b, Using One-way ANOVA*.

* *p ≦0.05*,

** *p ≦0.01, and*

*** *p ≦0.001*.

**Table 2:** Neuropsychiatric inventory.

**Variables**	**CU (*n* = 47)**	**prAD (*n* = 40)**	**prDLB (*n* = 30)**	***x* ^**2**^**	***p*-value**
Delusions	0 (0.00%)	9 (22.50%)	3 (10.00%)	11.89	0.003^**^
Hallucinations	0 (0.00%)	4 (10.00%)	4 (13.33%)	6.07	0.048^*^
Agitation/Aggression	0 (0.00%)	10 (25.00%)	3 (10.00%)	13.73	0.001^***^
Depression/Dysphoria	5 (10.6%)	14 (35.00%)	4 (13.33%)	9.14	0.010^**^
Anxiety	3 (6.38%)	12 (30.00%)	4 (13.33%)	9.11	0.011^*^
Elation/Euphoria	1 (2.13%)	0 (0.00%)	0 (0.00%)	1.50	0.472
Apathy/Indifference	0 (0.00%)	5 (12.50%)	1 (3.33%)	7.21	0.027^*^
Disinhibition	2 (4.26%)	7 (17.50%)	1 (3.33%)	6.25	0.044^*^
Irritability/Lability	1 (2.13%)	15 (37.50%)	3 (10.00%)	21.03	0.000^***^
Motor Disturbance	4 (8.51%)	13 (32.50%)	2 (6.67%)	11.86	0.003^**^
Nighttime Behaviors	11 (23.40%)	12 (30.00%)	14 (46.67%)	4.66	0.097
Appetite/Eating	3 (6.38%)	8 (20.00%)	5 (16.67%)	3.70	0.157

*Data are presented as number (percentage)*.

*CU, cognitively unimpaired; prAD, prodromal AD; prDLB, prodromal DLB; NPI, Neuropsychiatric Inventory*.

*Using Chi-square*.

* *p ≦0.05*,

** *p ≦0.01, and*

*** *p ≦0.001*.

Regarding neuroimaging studies, 36 prAD patients underwent brain ECD SPECT, and 25 showed decreased perfusion in the parietal association area, posterior cingulate, and posterior precuneus. Fourteen prDLB underwent brain SPECT showing decreased perfusion in the occipital area, which is a supportive index for a diagnosis of DLB. Among the 28 prDLB who received TRODAT examination, all showed markedly decreased bilateral striatal DAT availability, with 10 more severe on the left side, 6 on the right, and 12 equally severe on both sides.

### Time Perception Test

**Figure 2:**
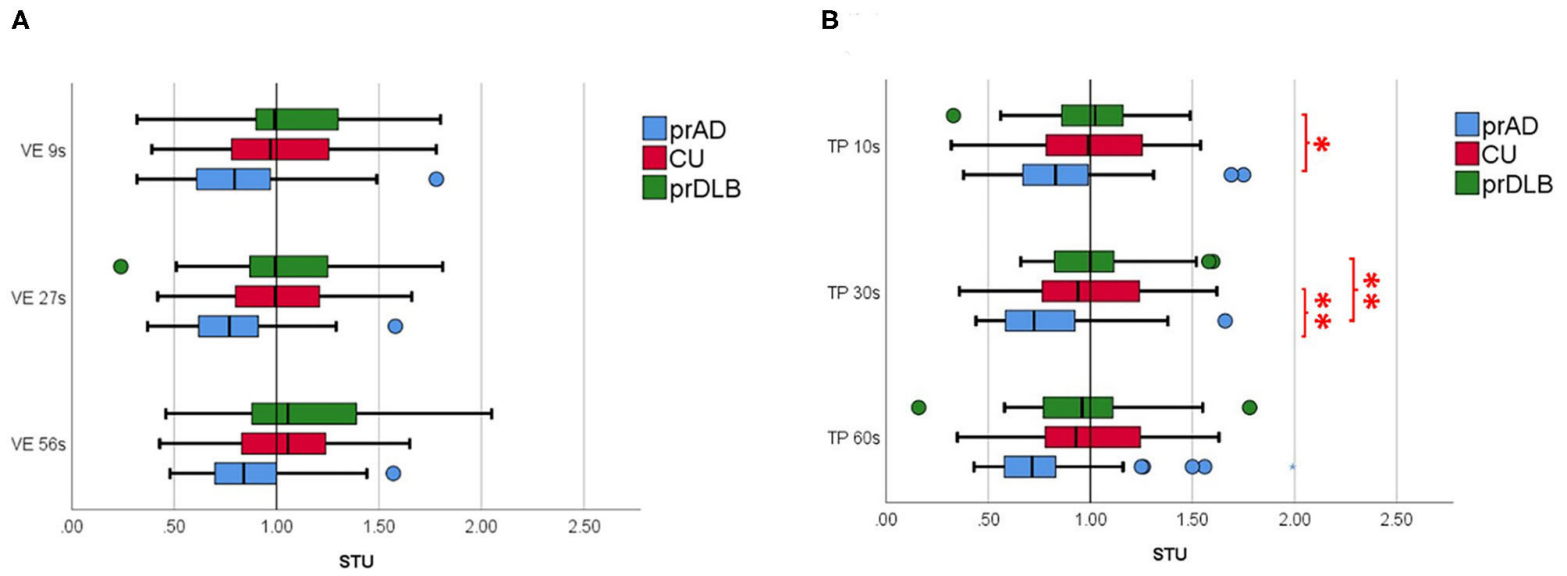
The subjective temporal unit (STU) of the participants in **(A)** verbal time estimation (VE) and in **(B)** time interval production (TP) in different time intervals. prAD, prodromal AD dementia; CU, cognitively unimpaired; prDLB, prodromal dementia with Lewy bodies (*p < 0.05, **p < 0.01).

In the VE test, at interval 9 s, the CV of prAD is higher than that of the CU/ prDLB (*F* = 6.45, *p* = 0.002), and at interval 27 s, the CV of prAD is higher than that of CU (*F* = 4.28, *p* = 0.016). At interval 56 s, no difference was found among the three groups (*F* = 2.81, *p* = 0.064). CV was known as a measure of timing variability. The larger the CV value, the more timing imprecision. No difference was detected in ATE, ABS, and STU among the three groups, see Table 3 and Figure 2A.

**Table 3:** Verbal estimation test.

**Variables**	**CU (*n* = 47)**	**prAD (*n* = 40)**	**prDLB (*n* = 30)**	***F***	***p*-value**	***post-hoc***
**9 s**						
ATE (second)	10.62 (4.71)	12.61 (5.45)	10.10 (7.84)	1.90	0.155	
ABS (second)	3.37 (3.66)	4.76 (4.54)	3.26 (7.22)	1.06	0.351	
CV	0.11 (0.08)	0.22 (0.18)	0.13 (0.14)	6.45	0.002^**^	prAD > prDLB = CU
STU (second)	1.00 (0.35)	0.95 (0.70)	1.10 (0.35)	0.81	0.448	
**27 s**						
ATE (second)	30.36 (12.60)	36.07 (13.48)	29.11 (17.95)	2.49	0.087	
ABS (second)	9.10 (9.28)	12.12 (10.75)	9.07 (15.59)	0.89	0.413	
CV	0.07 (0.07)	0.12 (0.11)	0.08 (0.09)	4.28	0.016^*^	prAD >CU
STU (second)	1.03 (0.37)	0.89 (0.48)	1.12 (0.41)	2.54	0.083	
**56 s**						
ATE (second)	61.80 (24.12)	68.93 (21.98)	55.89 (23.46)	2.76	0.067	
ABS (second)	17.56 (17.37)	19.78 (15.96)	15.70 (17.35)	0.51	0.602	
CV	0.06 (0.05)	0.10 (0.08)	0.11 (0.13)	2.81	0.064	
STU (second)	1.07 (0.40)	0.97 (0.65)	1.16 (0.40)	1.22	0.229	

*Data are presented as mean (standard deviation). Post-hoc analysis by Scheffe's test*.

*CU, cognitively unimpaired; prAD, prodromal AD; prDLB, prodromal DLB; ATE, average time estimation; ABS, absolute error score; CV, coefficient of variance; STU, subjective temporal unit*.

*Using One-way ANOVA*.

* *p ≦0.05*,

** *p ≦0.01*.

In the TP test, the ATE of prDLB is higher than that of prAD at intervals 10 s (*F* = 4.20, *p* = 0.017), 30 s (*F* = 5.49, *p* = 0.005), and 60 s (*F* = 4.78, *p* = 0.010); the ATE of CU is higher than that of prAD at interval 60 s. Although significant difference exists in ATE, ABS is the main indicator of accuracy. ABS was calculated by taking the difference between the participant's estimation and the target interval, without considering the sign. If a particular participant tended to error in both directions of over-and underestimation, the average error would not toward zero. The results show no significant difference in ABS among the three groups. Notably, at intervals 10 s (*F* = 4.21, *p* = 0.017) and 30 s (*F* = 5.76, *p* = 0.004), the STU of prDLB was larger than 1.0 s, while that of prAD was <1.0 s and differences were detected between these two groups, see Table 4 and Figure 2B. The proportions of people whose STU is either more than 1.0 s or <1.0 s, indicating under-or overestimation of time, are shown in Tables 5, 6. In CU, those with under-or overestimation is 50% for each and this was also the case in prDLB, while 70–80% of prAD tended to overestimate time. These results indicate that prDLBs have a tendency to underestimate time, while prADs overestimate.

**Table 4:** Time interval production test.

**Variables**	**CU (*n* = 47)**	**prAD (*n* = 40)**	**prDLB (*n* = 30)**	***F***	***p*-value**	***post-hoc***
**10 s**						
ATE (second)	10.04 (3.16)	9.00 (3.59)	11.74 (5.21)	4.20	0.017^*^	prDLB > prAD
ABS (second)	2.63 (1.77)	3.12 (2.31)	3.46 (4.25)	0.87	0.424	
CV	0.11 (0.11)	0.18 (0.21)	0.13 (0.13)	2.36	0.099	
STU (second)	1.00 (0.32)	0.90 (0.36)	1.17 (0.52)	4.21	0.017^*^	prDLB > prAD
**30 s**						
ATE (second)	28.71 (9.93)	24.09 (8.48)	31.94 (11.90)	5.49	0.005^**^	prDLB > prAD
ABS (second)	8.15 (5.74)	9.32 (4.48)	8.26 (8.75)	0.42	0.655	
CV	0.08 (0.12)	0.09 (0.09)	0.08 (0.06)	0.17	0.846	
STU (second)	0.98 (0.33)	0.80 (0.28)	1.06 (0.40)	5.76	0.004^**^	CU = prDLB > prAD
**60 s**						
ATE (second)	58.59 (19.92)	47.05 (19.82)	60.67 (22.94)	4.78	0.010^*^	CU = prDLB > prAD
ABS (second)	16.46 (11.05)	21.22 (10.22)	16.20 (16.46)	2.03	0.137	
CV	0.05 (0.04)	0.11 (0.19)	0.09 (0.09)	2.05	0.133	
STU (second)	0.98 (0.33)	0.95 (0.76)	1.01 (0.38)	0.12	0.891	

*Data are presented as mean (standard deviation). Post-hoc analysis by Scheffe's test*.

*CU, cognitively unimpaired; prAD, prodromal AD; prDLB, prodromal DLB; ATE, average time estimation; ABS, absolute error score; CV, coefficient of variance; STU, subjective temporal unit*.

*Using One-way ANOVA*.

* *p ≦0.05*,

** *p ≦0.01*.

**Table 5:** Verbal estimation test numbers of people STU lower or higher than 1 s.

**Variables**	**CU (*n* = 47)**	**prAD (*n* = 40)**	**prDLB (*n* = 30)**	***x* ^**2**^**	***p*-value**
**9 s**					
STU > 1 s	23 (48.94%)	9 (22.50%)	15 (50.00%)	7.91	0.019^*^
STU <1 s	24 (51.06%)	31 (78.50%)	15 (50.00%)		
**27 s**					
STU > 1 s	24 (51.06%)	10 (25.00%)	16 (53.33%)	7.85	0.020^*^
STU <1 s	23 (48.94%)	30 (75.00%)	14 (46.67%)		
**56 s**					
STU > 1 s	26 (55.32%)	11 (17.50%)	16 (53.33%)	7.80	0.020^*^
STU <1 s	21 (44.68%)	29 (82.50%)	14 (46.67%)		

*Data are presented as number of people (percentage)*.

*CU, cognitively unimpaired; prAD, prodromal AD; prDLB, prodromal DLB; STU, subjective temporal unit*.

*Using Chi-square;*

* *p ≦0.05*.

**Table 6:** Time interval production test numbers of people STU lower or higher than 1 s.

**Variables**	**CU (*n* = 47)**	**prAD (*n* = 40)**	**prDLB (*n* = 30)**	***x* ^**2**^**	***p*-value**
**10 s**					
STU > 1 s	22 (46.81%)	11 (17.50%)	19 (63.33%)	9.09	0.011^*^
STU <1 s	25 (53.19%)	29 (72.50%)	11 (36.67%)		
**30 s**					
STU > 1 s	20 (42.55%)	9 (22.50%)	15 (50.00%)	6.34	0.042^*^
STU <1 s	27 (57.45%)	31 (78.50%)	15 (50.00%)		
**60 s**					
STU > 1 s	19 (40.43%)	8 (20.00%)	10 (33.33%)	4.22	0.121
STU <1 s	28 (59.57%)	32 (80.00%)	20 (66.67%)		

*Data are presented as number of people (percentage)*.

*CU, cognitively unimpaired; prAD, prodromal AD; prDLB, prodromal DLB; STU, subjective temporal unit*.

*Using Chi-square;*

* *p ≦0.05*.

## Discussion

We used two tests to investigate how time perception was present in a group of people with prAD or prDLB and found a distinctive pattern in these two most common types of primary degenerative dementia. In the VE test, when target time intervals were short (9 and 27 s), prAD performed less precisely with larger CV compared to both CU and prDLB or CU only. When the target time interval was longer, the differences disappeared. These findings are parallel with previous studies. For example, AD participants have shown significant imprecision and variability in their time estimates (25). AD participants may also be more variable in estimating time intervals of similar lengths (24) and the most frequent error AD patients make is overestimation, especially on attentional tasks (26). As in this study, the prAD had poorer performance on the items of CASI in recent memory, orientation, and list generation compared with CU/DLB, which supports the aforementioned.

Another important finding of this study is a different trend of STU in prDLB and prAD. In the TP test, the STU of prDLB at interval 10 s was notably higher than that of prAD, while the CU showed no difference from prAD or prDLB. In addition, a higher proportion of prDLB had a STU more than 1.0 s at interval 10 s, indicating underestimation of time intervals. This difference is also seen between prDLB and prAD/CU in TP at interval 30 s. Based on the above findings, prAD showed a different behavioral pattern from prDLB in that the former tended to be less precise in the VE and overestimate in the TP, particularly with shorter time intervals.

Although the psychological processes needed to carry out TP and VE involve attention, memory, and executive functions (27) for both, in performing a TP test, a subject may rely more on their own mental control and more actively produce time intervals with greater confidence. The VE, on the other hand, more uncertainty existed as a subject was passively to perceive the time duration and this could need more sustained attention. Under such conditions, one might exhibit symptoms of anxiety and become restless before responding. This task-induced anxiety may be reflected in the higher proportion of STU larger than 1.0 s in the group prDLB in the VE, although not reaching statistical significance, see Table 5. In both tests, differences shown in shorter intervals may no longer exist at longer intervals. It has been reported that working memory and long-term memory play an essential role in time estimation, and intervals under 30 s require working memory, while those longer than 30 s need long term memory (24, 28, 29). In this study, poorer performance of prAD on the items of CASI might have affected the outcome of short intervals and not have affected longer intervals.

It is known that people with Parkinson's disease, a disease that shares the same changes in the brain and very similar pathological symptoms with DLB, underestimate time intervals in a VE task (30). In our study, all the 28 prDLB who performed TRODAT showed reduced dopamine transporter availability, indicating a presynaptic Parkinsonism. The internal clock may rely on dopamine signals to adjust the internal time. Dopaminergic neurons in the substantia nigra, mainly terminating in dopamine D2 receptors, work as pacemaker units, and pulses from these neurons accumulate in the dorsal striatum (31, 32). As dopamine is reduced, fewer pulses that are transferred into time perception accumulate. This may result in underestimation of accurate time perception. In addition, people with DLB more often had symptom of anxiety than AD (33), and previous research shows anxiety is associated with temporal underestimation, which is compatible with our finding (34). On the contrary, it is not known whether overestimation in prAD would disturb their memory or not. It is also unclear whether the cause of this distortion is attention, memory, or executive function. To answer these questions, more research is needed.

One of the advantages of this study is the inclusion of both prDLB and prAD. Without it, the distinct patterns and differences between the two groups cannot be shown. This may also open a window to learn about the potential discriminators for these most important primary degenerative dementias in both diagnosis and early intervention. It was quite unexpected that no differences were detected in the accuracy at any intervals on either VE or TP among the three groups. This may just reflect the reality of the prodromal stage of dementia. One might suggest adopting time estimation with longer time intervals, such as several minutes, hours, or even days. However, this may raise more confounders that cannot be controlled easily. One might also suggest inviting patients at a more advanced stage of dementia. People at this stage, however, usually cannot grasp the instructions for more complicated experiments and unexpected conditions may arise.

Limitations of the current study are addressed here. Controlling participants' emotion during testing is usually impractical. Previous studies showed intense emotions may lead to serious overestimation or underestimation. In this study, when a participant got too emotional, we tried to comfort them first. When it got out of control, we terminated the test. As mentioned, the prodromal stage contains patients of varying severity with different kinds of clinical symptoms. Diversity may exist in the deficit of neural networks or cognition defects essential for time perception in prAD or prDLB and this may explain why some patients in the prodromal dementia stage remain normal in their time perception. Participants' medication status might also affect the study's outcome. Although more prAD were using cholinesterase inhibitor than prDLB in our study, we have yet found any research reports regarding the effect of cholinesterase inhibitors on time perception. Future studies are needed to answer this question. In response to the SARS-Cov-2 pandemic, many prodromal stage patients declined to join this study and we invited several patients' spouses as controls. Thorough neuropsychological tests before the study procedure, they were shown to be cognitively unimpaired. Moreover, patients who came to join this study might be in a better condition and had more extroverted personalities. These situations might have affected the results of this research.

Although the results of this study gave us an insight into the time perception in PrAD and PrDLB, some matters need to be more cautious when interpreting the results. As the recent pandemic affected the number of patients we recruited, further study should recruit more participants. With more participants recruited, sex, age, and other confounders can also be well-controlled. Further research can extend the time interval to more than 1 minute to explore differences among the three groups on a larger time scale. Meanwhile, future research can divide prAD and prDLB into groups based on cognitive deficits and severity and test the time perception of each group separately for a more in-depth understanding of time perception. Finally, a longitudinal follow-up study can be conducted to see whether more pronounced timing deficits would exhibit as the disease progresses.

**Figure 3:**
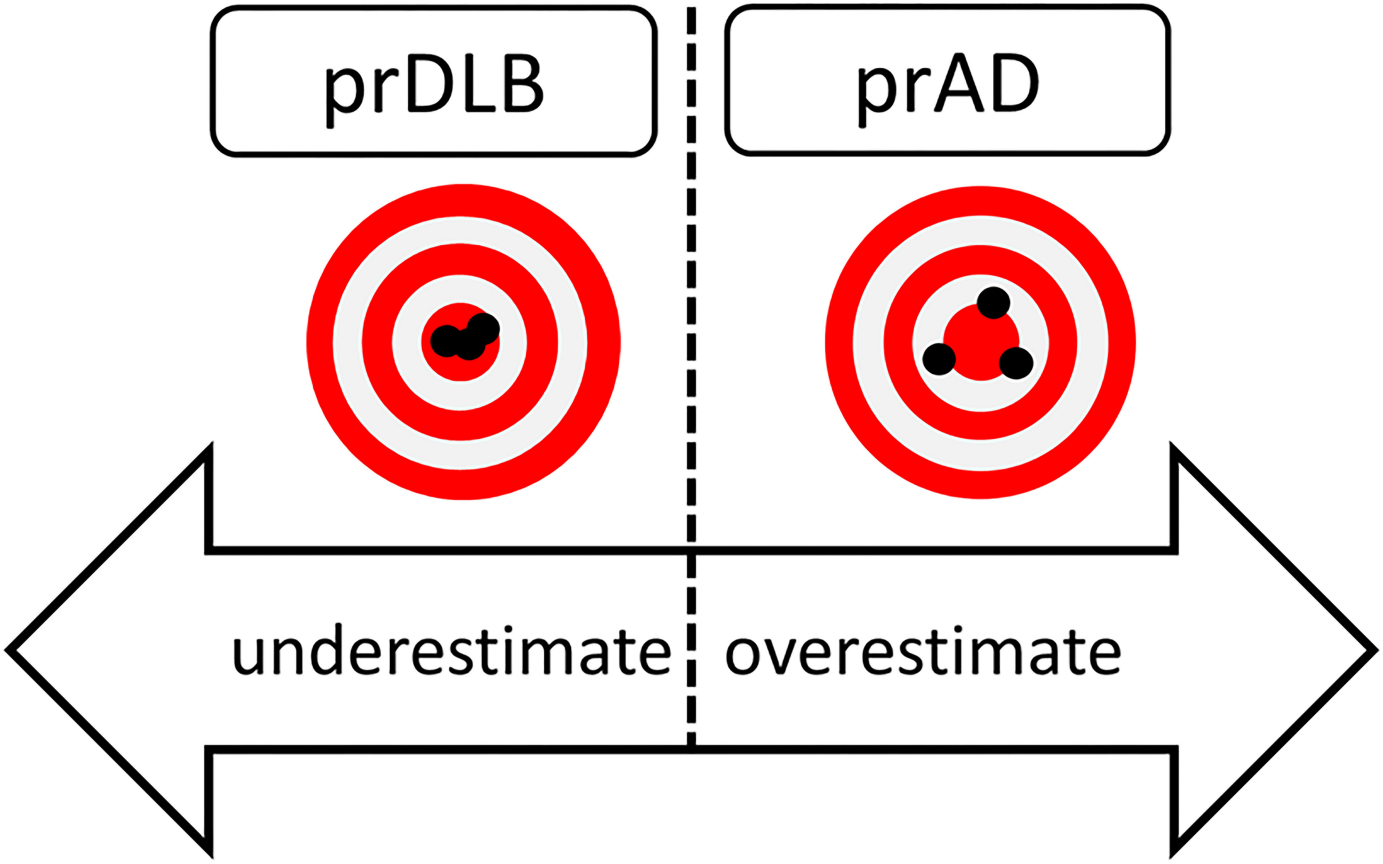
Hypothetical figure of the results of time perception in prDLB and prAD. PrDLB tended to underestimate time while prADs were less precise and tended to overestimated time. prAD, prodromal AD dementia; prDLB, prodromal dementia with Lewy bodies.

In summary, this study shows that most prAD were less precise and overestimated the time elapsed compared to prDLB, while prDLB tended to underestimate time interval, see Figure 3. How significant these differences are in influencing daily life deserves more research.

## Data Availability Statement

The raw data supporting the conclusions of this article will be made available by the authors, without undue reservation.

## Ethics Statement

The studies involving human participants were reviewed and approved by Medical Ethics Review Committee of the National Cheng Kung University Hospital. The patients/participants provided their written informed consent to participate in this study.

## Author Contributions

M-CP and C-JY were responsible for the study concept. M-CP enrolled subjects, performed clinical diagnosis for all participants, and reviewed and revised the manuscript. C-JY carried out the experiments, conducted the statistical analysis, and prepared the manuscript. M-CP, S-YF, and C-JY discussed the results and contributed to the final manuscript. All authors contributed to the article and approved the submitted version.

## Funding

This study was in part sponsored by a grant MOST109-2514-S-006-005 to M-CP.

## Conflict of Interest

The authors declare that the research was conducted in the absence of any commercial or financial relationships that could be construed as a potential conflict of interest.

## Publisher's Note

All claims expressed in this article are solely those of the authors and do not necessarily represent those of their affiliated organizations, or those of the publisher, the editors and the reviewers. Any product that may be evaluated in this article, or claim that may be made by its manufacturer, is not guaranteed or endorsed by the publisher.
